# Editorial: Insights in PET and SPECT: 2023

**DOI:** 10.3389/fnume.2023.1342672

**Published:** 2023-12-21

**Authors:** Mario Petretta, Carmela Nappi, Alberto Cuocolo

**Affiliations:** ^1^ Department of Nuclear Medicine, IRCCS SYNLAB SDN, Naples, Italy; ^2^ Department of Advanced Biomedical Sciences, University of Naples Federico II, Naples, Italy

**Keywords:** nuclear medicine, SPECT, PET, metabolic imaging, molecular imaging

**Editorial on the Research Topic** Insights in PET and SPECT: 2023

It is a pleasure and an honor for us to introduce the Research Topic *Insights in PET and SPECT: 2023*, published in the PET and SPECT section of Frontiers in Nuclear Medicine. The development of tomographic technologies such as single photon emission computed tomography (SPECT) and positron emission tomography (PET) began in the 1970s. Since then, PET and SPECT have established themselves as extremely useful methods in the clinical field thanks to their high sensitivity, deep penetration capacity and an increasingly wider range of radiopharmaceuticals (1, 2). Both techniques are currently used in many diseases, including Alzheimer's, Parkinsons, and other nervous system diseases, cardiovascular diseases, and chronic inflammatory disorders. In oncology, anatomical imaging techniques may detect tumors with a diameter of one centimeter or more, which already contain more than a billion cells, while functional imaging PET and SPECT can detect small tumors up to one millimeter (3). This Research Topic includes five Original Contributions, one Systematic Review, and two Case Reports, covering several aspects of a spectrum of disease.

## Phantom study to assess stability of PET radiomic features

Radiomics aims to enhance the potential of medical imaging through mathematical extraction and analysis of the spatial distribution of signal intensities and pixel interrelationships. Radiomic features quantify textural information as promising metrics for diagnostic and prognostic evaluation of several diseases, including cancer. However, variability in hardware, software, respiration, and other variables may impact the stability of PET images radiomic features and their utility (4). Alsyed et al. present data from a phantom study evaluating the ability of radiomics features to distinguish PET image regions with different uptake patterns based on different image reconstructions settings. The study identified 15 features stable to reconstruction parameters and showing statistically significant differences in the presence of different levels of phantom designed spatial heterogeneity. The findings encourage the design of further studies involving clinical data using a similar approach to evaluate the potential of translational application of the observed results.

## Whole-body CZT camera in a PET-like utilization

Quantification of bone SPECT images is useful for the differential diagnosis of several diseases, such as osteomyelitis, osteonecrosis, degenerative bone lesions and primary and metastatic cancers (5). Moreover, quantitative SPECT images allow to track longitudinal spontaneous (e.g., due to progression of disease) and treatment induced changes. Previous quantitative bone SPECT studies have been performed using conventional Anger cameras; however, this approach can't get timely pictures showing images that display whole-body recordings using a standardized uptake value (SUV) scale. To overcome these limitations, Bahloul et al. aimed to determine whether such PET-like images may be obtained with a high-speed cadmium-zinc-telluride (CZT)-SPECT/CT system, with a further application for the longitudinal monitoring of vertebral fractures, using a new high-speed whole-body CZT camera. This camera not only enhances images quality but also reduces SPECT recording times to <20 min, as currently required for PET imaging (6). With this CZT-SPECT/CT system, the authors were able to obtain fast 3D acquisitions and high-quality images of vertebral fractures displayed on a reliable SUV scale, similarly to what is achieved and recommended for PET imaging.

## Prognostic value of PET parameters in patients with head and neck squamous cell carcinoma undergoing immune checkpoint inhibitor therapy

In recent years, immune checkpoint inhibitors have proven to be a valid therapeutic strategy in patients with recurrent and/or metastatic head and neck cancer (HNSCC). However, further research is needed for the definition of optimal treatment schemes and above all to identify prognostic biomarkers useful for the appropriate selection of patients (7). Kwon et al. evaluated the prognostic value of basal metabolic features of ^18^F-FDG PET/CT in patients with HNSCC treated with immune checkpoint inhibitors. The authors also assessed the correlations between these features and immunohistochemical biomarkers. The authors found a negative correlation between programmed cell death-ligand 1 expression and metabolic tumor volume. Moreover, the progression-free survival was inversely related to metabolic tumor volume and total lesion glycolysis. These data support the prognostic potential of ^18^F-FDG PET/CT in HNSCC patients treated by immunotherapy.

## Regional coronary blood flow by CZT cameras for evaluation of coronary artery disease

Stress SPECT myocardial perfusion imaging is an established technique for diagnostic and prognostic purposes in patients with suspected or known coronary artery disease (CAD) (8). The introduction of dedicated gamma cameras with CZT semiconductor detectors has enabled significant progress in the clinical evaluation of heart disease due to increased photon sensitivity and spatial resolution compared to the conventional SPECT system (9, 10). In particular, CZT cameras allow noninvasive quantification of an important physiologic measure, such as myocardial flow reserve (MFR). Lima et al. aimed to compare myocardial perfusion SPECT with or without MFR evaluation for the detection of obstructive CAD. The authors found that the addition of MFR data to perfusion data improved the detection of CAD over perfusion abnormalities by CZT-SPECT imaging alone, especially in patients with multivessel CAD. A regional MFR of 2.0 provided the best trade-off between sensitivity and specificity for identifying obstructive CAD.

## Disparities of PET/CT use in gastro-esophageal cancer

Esophagus or gastroesophageal junction cancers have a high global incidence and mortality rates (11). Imaging techniques, in particular ^18^F-FDG-PET/CT, have significantly enhanced the accuracy of clinical staging. In general, while staging of locoregional disease is usually best done with a combination of CT and endoscopic ultrasound, any metastases are better recognized with ^18^F-FDG-PET/CT (12). Currently, PET/CT is recommended in multiple guidelines for staging these types of cancer; however, in clinical settings the use of this imaging technique is highly variable. Therefore, health disparities exist due to multiple and complex reasons (13). Gupta et al. report the results of an analysis performed on data obtained from the Population Registry of Esophageal and Stomach Tumors of Ontario and Ontario Health. Notably, women underwent less PET/CT than men, and patients with esophagus cancer were referred more than patients with gastroesophageal junction cancer. Other differences in demographic and clinical variables in receipt of PET/CT were found.

## Diagnostic performance of ^18^F-FDG PET/Ct vs. PET/MRI for the diagnosis of colorectal liver metastasis

Detection of liver metastases in colorectal cancer (CRC) patients is complicated and still represents a major clinical challenge (13). In CRC patients, the liver is a frequent site of metastatic disease. Often, at the time of detection metastases are limited to the liver, and available treatment options, such as complete resection of lesions, can be curative and beneficial to survival (14). These considerations underline the importance of correct staging at baseline and during follow-up of patients with CRC, in order to be able to choose a personalized therapeutic approach. Different diagnostic imaging techniques are available for CRC staging or restaging. Further progress has been made with the introduction of integrated imaging techniques, such as hybrid PET/CT and hybrid PET/magnetic resonance imaging (MRI). However few studies quantitatively compared the relative clinical performance of these two approaches. Miao et al. performed a systematic review and meta-analysis on this topic, searching PubMed, Embase, and Web of Science databases for eligible articles until November 2022. Finally, a total of 21 articles evaluating the diagnostic performance for colorectal liver metastasis, 16 for PET/CT and 5 for PET/MRI, were included. ^18^F-FDG-PET/CT and ^18^F-FDG-PET/MRI show similar performance in detecting colorectal liver metastasis. However, as noted by the authors, pathological results were not available for all patients in the included studies and PET/MRI results were derived from studies with small sample sizes. Therefore, further larger prospective studies on this topic are welcome.

## Case reports

This Research Topic includes two Case Reports. Cortés Mancera et al. describe a 67-year-old male patient diagnosed with prostate adenocarcinoma with a clinical picture of muscle weakness in the lower limbs and other neurological symptoms and signs. In this patient, ^18^F-FDG-PET/CT imaging was able to demonstrate that amyotrophic lateral sclerosis, prostate cancer and progressive supranuclear palsy may coexist in the same patient. Finally, Nearchou et al. describe the first case utilizing ^18^F-prostate specific membrane antigen (PSMA)-PET/CT and 99mTc-sulphur colloid SPECT to detect intraabdominal splenosis, highlighting the high potential of nuclear medicine in such trivial cases.
